# Early and Mid-Term Outcomes of Isolated Type 2 Endoleak Refractory to an Embolization Procedure

**DOI:** 10.3390/jcm14020502

**Published:** 2025-01-14

**Authors:** Francesca Miceli, Ada Dajci, Alessia Di Girolamo, Piergiorgio Nardis, Marta Ascione, Rocco Cangiano, Roberto Gattuso, Antonio Sterpetti, Luca di Marzo, Wassim Mansour

**Affiliations:** 1 Vascular and Endovascular Surgery Division, Department of General Surgery and Surgical Specialties, Policlinico Umberto I, “Sapienza” University of Rome, Viale del Policlinico 155, 00161 Rome, Italy; francesca.miceli@uniroma1.it (F.M.); ada.dajci@uniroma1.it (A.D.); marta.ascione@uniroma1.it (M.A.); rocco.cangiano@uniroma1.it (R.C.); roberto.gattuso@uniroma1.it (R.G.); antonio.sterpetti@uniroma1.it (A.S.); luca.dimarzo@uniroma1.it (L.d.M.); wassim.mansour@uniroma1.it (W.M.); 2 Radiology Department, Policlinico Umberto I, “Sapienza” University of Rome, Viale del Policlinico 155, 00161 Rome, Italy; p.nardis@gmail.com

**Keywords:** endoleak, AAA, EVAR, refractory endoleak type II, complication, endograft explantation, endovascular technologies, vascular and endovascular medicine, endovascular surgery

## Abstract

**Introduction:** A type 2 endoleak (EL2) remains the most prevalent complication of endovascular aortic repair (EVAR) for an abdominal aortic aneurysm (AAA). **Methods:** We conducted a retrospective, single-center analysis, including patients who underwent embolization for an isolated EL2 after EVAR. The study population was stratified into two groups: Group A, consisting of patients whose EL2 resolved after the first embolization procedure, and Group B, consisting of those with refractory EL2 (rEL2). The indication for EL2 treatment was aneurysmal sac growth amounting to >10 mm from the index EVAR. The indications for endograft explantation were the absence of high comorbidities and persisting aneurysmal sac expansion. Those with high comorbidities were subjected to another endovascular procedure or a conservative approach, the latter being preferred. The primary endpoint was EL2 resolution. The secondary endpoints were mid-term outcomes in terms of aneurysmal sac shrinkage, stability and expansion rates, and aneurysm-related complications. **Results:** Among 57 patients, 19 patients (33.3%) showed signs of EL2 after the first embolization, whereas 38 (66.6%) presented rEL2. Of these, 14 (36.8%) presented significant aneurysmal sac expansion: 8 patients underwent a secondary embolization, while an open conversion was performed in the remaining 6 patients (42.8%), 4 of whom, in an elective setting, showed a complete resolution of EL2, while 2 patients treated in an urgent setting died from a ruptured aneurysm. Among the patients treated with a secondary embolization, only 2 patients presented EL2 resolution, while the other 6 patients (75%) showed rEL2. Out of the 38 patients with rEL2, 24 patients did not undergo further interventions; of these, 11 (45.8%) presented sac expansion, and 16% developed type IA EL. **Conclusions:** A strict follow-up and possibly a more aggressive treatment should be considered in an elective setting for patients with rEL2.

## 1. Introduction

The use of endovascular procedures to treat abdominal aortic aneurysms (AAAs) has increased exponentially over the last 20 years. Presently, EVAR is regarded as the gold-standard technique due to its associated lower perioperative mortality and morbidity, as well as a shorter hospital stay [1]. However, as demonstrated by several randomized controlled trials (RCTs) [2,3,4,5], except for the OVER trial [6], EVAR has a clear benefit with respect to open surgical repair only in the early period, without further differences in the mid- and long-term follow-ups. A recent meta-analysis has shown that EVAR is associated with a higher risk of AAA-related death, reinterventions, and rupture after 8 years of follow-up [7]. Secondary interventions are mainly required due to the occurrence of endoleaks (ELs), the most common complication after EVAR, occurring in up to 44% of cases within 6 months of EVAR [8]. EL2 is mainly considered to be a benign condition since it is characterized by a low-pressure retrograde flow into the aneurysmatic sac from aortic branches, mainly lumbar arteries (LAs), the inferior mesenteric artery (IMA), accessory renal arteries [9], and the median sacral artery (MSA) [10]. Half the cases of EL2 resolve spontaneously within 6 to 12 months. However, if there is no resolution within this timeframe, they are defined as persistent endoleaks (pEL2). In these cases, in which the presence of pEL (>6 months) is associated with expansion of the AAA sac (>5 mm/year), reintervention should be considered [11]. Endovascular treatment options include the embolization of aortic vessel branches via coils, plugs, or tissue adhesives. In recent years, the use of liquid embolic materials, like Onyx, has become widespread [12]. These materials have some advantages over classic coils, such as the ability to migrate and fill the sac and the ability of the afferent and efferent branches to form a solid, non-compressible cast that can prevent EL recurrence [13]. The interest in preventive measures for avoiding pEL2 has been gradually increasing recently, as many reintervention strategies have not demonstrated an increased percentage of effectiveness. The aim of the present study is to evaluate the early and mid-term outcomes of an embolization procedure for isolated EL2 in a cohort of patients previously treated with EVAR at a single center.

## 2. Materials and Methods

We analyzed a single-center, retrospective database comprising patients who had been treated via EVAR for infrarenal AAA and then treated with a redo endovascular embolization procedure for isolated EL2. We also included patients treated with EVAR at outside centers. All patients requiring reintervention for isolated EL2 were retrospectively analyzed from a prospectively collected database. We excluded from the study all patients for whom computed tomography angiography (CTA) images captured before EVAR were unavailable, patients treated using approaches outside the devices’ instructions for use (IFU) or with endovascular aneurysm sealing (EVAS), and patients who presented with EL2 associated with other types of EL.

We stratified the entire study cohort into two groups: Group A included patients for whom EL2 resolved after the first embolization procedure, whereas Group B included patients with rEL2 after the first embolization procedure. At our center, indications for the EVAR procedure are based on the major international guidelines [14]. A careful clinical history was collected, and a physical examination was performed. Clinical comorbidities were evaluated prior to the performance of any procedures. After EVAR, the follow-up protocol included physical examination with abdominal Doppler Ultrasound (DUS) at 30 days, CTA at 30 days, and thereafter DUS yearly, unless complications are detected via DUS. In the case of EL2 with sac expansion (>10 mm), according to the latest ESVS guidelines [14], patients were scheduled to undergo endovascular procedures via aortic sac embolization and/or aortic side branch occlusion. However, patients with EL2 responsible for persisting aneurysmal sac expansion 6 months after redo endovascular procedures, defined as “refractory” (rEL2), underwent further endovascular procedures or open conversion. Patients with comorbidities underwent a further endovascular procedure, whereas indications for surgical conversion were based on clinical conditions and comorbidities. Patients with a lower surgical risk underwent aortic endograft explantation in an elective setting. Patients with abdominal pain or a ruptured aneurysm underwent open conversion in an urgent setting.

Open conversions were performed via median laparotomy with partial or complete removal of the endograft. All identified feeding vessels were ligated or sutured, and an aortic or aortoiliac reconstruction with a Dacron graft was performed. Patients with an endograft with a suprarenal fixation underwent partial endograft explantation, leaving the suprarenal fixation stent in situ. The primary outcome of the study was to evaluate the fate of EL2 after the first embolization procedure. Secondary outcomes were the need for further endovascular reintervention after the first embolization for refractory EL2, open conversion and AAA-related mortality. The demographic data of each patient was analyzed, together with the anatomical and technical characteristics of all procedures.

### Statistical Analysis

Continuous variables are given as mean and standard deviation. Categorical variables are given as a number (percentage). Analysis of the differences between the two groups was performed using the chi-squared test or Fisher’s exact test, where appropriate, for categoric variables. A *p*-value of <0.05 was considered statistically significant. All analyses were calculated using SPSS version 26 (IBM Corp, Armonk, NY, USA).

Ethical approval was waived due to the retrospective nature of the study, according to our Ethics Committee. Informed consent was obtained from all subjects involved in the study.

## 3. Results

From January 2017 to September 2024, 911 endovascular abdominal aortic aneurysm exclusion procedures were performed in our tertiary University Hospital.

Of this group, 203 (22.28%) had EL2 at the CTA 30 days after EVAR. A total of 132 (65%) had a spontaneous resolution of EL2 at the subsequent follow-up. In contrast, 71 patients (35%) showed persistent EL2. A total of 26 patients were excluded from the study because they had EL2 associated with other types of endoleaks.

A total of 45 patients were included in the study, and we added 12 patients treated by EVAR from other centers. A total of 57 patients were therefore enrolled in the study. We stratified the cohort into two groups based on the absence or presence of rEL2 after the first embolization procedure. Group A included 19 (33.3%) patients with EL2 resolution after the first embolization procedure, whereas Group B included 38 (66.6%) patients with rEL2 after the first embolization procedure. Demographics and comorbidities data are presented in Table 1. The analysis of cardiovascular risk factors revealed a positive, statistically significant correlation between ischemic heart disease and the development of rEL2 (*p* < 0.002) (Table 1).

Considering the primary intervention in the two groups of patients: in Group A, 4 (23%) patients underwent a standard EVAR procedure, 7 (34%) an EVAR combined with sac embolization and 8 (42%) an EVAR with inferior mesenteric artery embolization. In Group B, 74% of cases underwent a standard EVAR procedure, 20% EVAR combined with sac embolization and 6% EVAR with IMA embolization.

The incidence of rEL2 was higher in the group of patients undergoing primary standard EVAR than in the group of patients undergoing additional procedures such as sac embolization and IMA embolization (*p* = 0.001).

Multivariate analysis showed that an association of EVAR and adjunctive procedures (IMA, lumbar and aneurysmal sac embolization) were all independently associated with a lower risk of occurrence of rEL2, while the preoperative aneurysm diameter of >65 mm at the first EVAR procedure, was a predictive independent factor of rEL2 (Table 2).

In Group A, the diagnosis of EL2 was found in 79% of cases (15 patients) at the first CTA, while in Group B, it was found in 92% (35 patients) after 6 months, with a mean of 192 ± 14 days.

Anatomical characteristics measured at CTA revealed that the maximum aortic transverse diameter, at all follow-up intervals, was higher in Group B (*p* = 0.0001) (Table 3).

All commercially available types of endograft at our center were used in this sample (Excluder and C3, W.L. Gore & Associates, Flagstaff, AZ, USA; Endurant II, Medtronic Inc., Santa Rosa, CA, USA; AFX, Endologix, Irvine, CA, USA; Incraft, Cordis, Fremont, CA, USA; Ovation, Endologix, Irvine, CA, USA) with no statistically significant differences between the two groups in our study.

By retrospectively analyzing the CTA images, we assessed the patency of the inferior mesenteric artery (IMA) and the number of patent lumbar arteries (LAs) in both groups of patients (Table 4). Patency of the IMA was found to have a statistically significant correlation with the group of patients with rEL2 (*p* = 0.01). We performed a comparative analysis between the two groups considering the variable of the number of patent lumbar couples and observed that a higher number of patent LA couples had a statistically significant correlation with the development of rEL2. (*p* < 0.0001) (Table 4).

Considering the second procedure for EL2, it emerged that in Group A, 15.7% of patients underwent embolization of the IMA, 5.26% underwent transarterial embolization of the aneurysmal sac, 42.1% underwent embolization of the LAs, 10.5% underwent sac and IMA embolization and 26.3% sac and LAs embolization. In Group B, 18.42% had undergone embolization of the IMA, 68.9% transarterial embolization of the sac, 5.26% embolization of the LAs, 5.26% sac and IMA embolization and 5.26% sac and LAs embolization.

rEL2 did not correlate statistically with the IMA embolization procedure, whereas there was a statistically significant correlation with the sac embolization procedure (*p* = 0.000082) and the LA embolization procedure (*p* = 0.00001) (Table 5).

A sub-analysis of the patients in Group B was performed. Of the 38 patients with rEL2, 14 (36.8%) presented with an aneurysm sac expansion of >5 mm at 6 months after the embolization procedure. Of these 14 patients, 8 underwent a second embolization procedure, namely a lumbar embolization: 2 patients showed resolution of rEL2, while 6 patients continued to present rEL2. The remaining 6 patients underwent open conversion with explantation of the aortic endograft and ligature of the LAs originating from the aneurysmal sac. Notably, 4 patients were treated electively with the complete resolution of rEL2, while 2 patients, treated urgently due to abdominal pain or rupture of the AAA, both died intraoperatively. Of the 38 patients with rEL2, 24 were not treated with a further procedure due to slow growth or stability of the aneurysmal sac. A more conservative approach was therefore preferred. However, during a mean follow-up at 37.5 months after the first embolization procedure (range, 1–108 months), out of 24 patients, 11 (45.83%) presented aneurysmal sac expansion, 5 (20.83%) sac shrinkage, and 8 (33.33%) sac stability.

A conservative approach was decided upon for the 11 patients with aneurysmal sac expansion because 8 patients presented a prohibitive risk for open surgery (4 patients with a recent history of myocardial infarction, 2 patients with a recent history of pneumonia, 2 patients with a neoplasm undergoing treatment) and 3 patients who refused further intervention.

**Figure 1:**
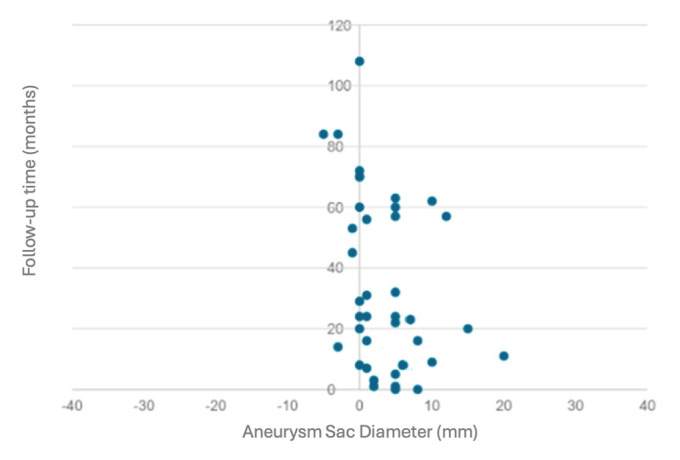
Scatter plot illustrating the change in aneurysmal sac diameter following the first embolization procedures over time. Each point represents the diameter change in millimeters at the recorded follow-up time in months for individual patients. Negative values indicate a reduction in sac diameter, whereas positive values represent an increase.

However, at the last follow-up of all the patients with rEL2 (38 patients), 25 (65.78%) presented aneurysmal sac expansion, while 13 patients (34.21%) had sac shrinkage (5 patients) or presented sac stability (8 patients) (Figure 1). Among the patients with sac expansion, 4 (16%) developed type IA EL after a mean follow-up of 42 months. Among those with type IA EL, two patients were treated with an aortic endograft explantation by performing an aortic reconstruction with a Dacron graft and suture of the LAs. The remaining two patients were treated with relining by custom-made, fenestrated EVAR (F-EVAR) due to a prohibitive risk for open surgery. All patients were treated in an elective setting with a 100% technical and clinical success rate.

## 4. Discussion

Endovascular treatment (EVAR) of AAAs has proven to be a viable alternative to open surgical treatment due to its minimally invasive approach.

Secondary intervention rates have been shown to be higher in patients undergoing endovascular procedures compared to those treated with conventional open repair [9]. Therefore, the Achilles heel of endovascular procedures is the long-term duration; consequently, close surveillance is mandatory to anticipate and treat complications, especially the occurrence of ELs.

Any type of EL accounts for 83% of indications for reintervention after EVAR, of which 65% are related to reintervention for persistent EL2 [15]. As is well known, the presence of EL2 is the most common complication after endovascular treatment of AAA, with an incidence of 25% [16], and previous studies have reported that 60% of EL2s resolved spontaneously within 6 months. From our experience, the incidence of EL2 was 22.28% at a 30-day CTA after an EVAR procedure. A total of 65% had a spontaneous resolution of EL2 at the subsequent follow-up. In contrast, 71 patients (35%) had persistent EL2.

Endovascular reinterventions for EL2 associated with expansion of the aneurysmal sac after EVAR have a lower impact on the stability or shrinkage of the aneurysm diameter.

In our analysis, we observed a 66.6% rate of refractory EL2 despite a previous endovascular correction procedure, and only 34.21% of cases experienced shrinkage or stability of the sac after the first embolization procedure. Notably, in our series, patients treated at the first redo endovascular procedure with direct transarterial embolization of the aneurysmal sac showed a higher risk of presenting rEL2 compared to those treated with IMA embolization or lumbar embolization (*p* = 0.000082).

65.78% of the cases presented an increase in the aneurysmal sac, with 16% evolving into EL IA. In a recent study, the group of patients with a stable sac diameter had a greater association with poly-district atherosclerotic manifestations. In terms of comorbidities, the estimated 12-year survival was 42.9% in the group with a stable sac and 65% in the group with sac shrinkage (*p* < 0.001), although there was no significant difference in terms of the absence of aneurysm-related death (97.3% vs. an estimated 95.4% at 12 years, *p* = 0.493) [17]. Patients with a stable sac had higher rates of rupture (2.1% vs. 0.6%, *p* = 0.035) and surgical conversion (2.1% vs. 0.6%, *p* = 0.035). The stable sac group had significantly higher rates of ELs of all types during FU (45.8% vs. 24%, *p* < 0.001). The estimated rates of freedom from reintervention at 12 years were 56.2% in the group with stability and 83.9% in the group with shrinkage, respectively (*p* < 0.001).

In our sample of patients, of those with shrinkage and stability of the sac, only 5 patients showed a reduction of the sac, whereas the other 8 patients showed stability of the sac. These patients should, therefore, not be considered to be a low-risk category, but rather as part of that 65.78% with an increased aneurysmal sac and thus considered to be high risk for further reinterventions and AAA-related complications.

EL2 appears to be a negative prognostic marker, but no effective preventive strategy exists. Some authors [18,19] suggest the preventive use of embolization of the IMA, lumbar, sacral arteries, or embolization of the sac to reduce the risk of EL2 incidence and its associated reinterventions. In our study, the incidence of rEL2 was higher in the group of patients undergoing primary standard EVAR than in the group of patients undergoing additional maneuvers, such as sac embolization and IMA embolization (*p* = 0.001).

In our study, analyzing the cardiovascular risk factors between the two groups, chronic ischemic heart disease was found to have a statistically significant association with the presence of rEL2. This finding could be an expression of the continued activity and release of pro-inflammatory cytokines by the residual aneurysmal sac. Many authors argue that endovascular exclusion of the abdominal aortic aneurysm transforms the aneurysmal pathology into a new, chronic condition [20,21]. The residual aneurysmal sac may undergo progressive remodeling of the composition of the extracellular matrix with consequent release into the circulation of products of its degradation, as well as continuous inflammatory processes, with consequent dysregulation of the levels of pro-inflammatory cytokines in the circulation [22].

In their attempt to categorize EL2 risk, Piazza and collaborators [23] classified patients as “at risk” if they met at least one of the following requirements: patency of an IMA of more than 3 mm, at least three lumbar artery pairs, two LA pairs plus a sacral artery, an accessory renal artery, or a patent IMA of any diameter. In our study, we observed that a higher number of patent LA couples had a statistically significant correlation with the development of rEL2, and the patency of the IMA was found to have a statistically significant correlation with the group of patients with rEL2.

Furthermore, our study has allowed us to identify the aortic aneurysm diameter at EVAR time as a predictive factor of rEL2. Moreover, patients who underwent isolated standard EVAR were more prone to developing rEL2 compared to those who underwent EVAR associated with adjunctive procedures. These findings highlight that in those with larger aneurysm diameter, an EVAR procedure with adjunctive endovascular procedures may be indicated, and a careful evaluation of anatomical characteristics should be performed to identify any lumbar artery or a patent IMA to be embolized since, in our series, the transarterial sac embolization, as a redo procedure, does not allow the resolution of the complication.

In a recent study, the estimated freedom from AAA expansion in the pEL2 group was 100%, 96.7%, 85.2% and 54.3% after 1, 2, 3 and 4 years of FU, respectively. In the control group without EL2, the freedom from AAA expansion was 100% after 1, 2, 3 and 4 years (*p* < 0.01) [24]. Therefore, it is known that pEL2 is associated with AAA expansion in approximately 70% of cases after 5 years of FU, which can be interpreted as a risk factor for rupture [25,26]. Our analysis during the follow-up period showed that most patients with rEL2 developed an increased aneurysmal sac, thus increasing the risk of AAA-related complications. Although not statistically significant, the presence of rEL2 is more strongly correlated with AAA-associated mortality. Notably, in our study, all patients with ruptured or symptomatic AAA treated with open conversion in an urgent setting died. This is in line with the fact that urgent interventions are more associated with mortality than those treated in the elective setting [27].

In the case of failure of endovascular EL2 correction procedures and evidence of aneurysmal sac growth, open conversion with preservation or explantation of the endoprosthesis must be considered, in accordance with new recommendation number 108 in the most recent European guidelines [14],

Some authors report that endograft explantation is associated with a high risk of peri- and postoperative mortality and morbidity [28]; however, other authors [29,30] report that there is no statistically significant difference in terms of mortality and morbidity between elective surgical conversion and primary surgical treatment for abdominal aortic aneurysm resection.

However, studies [21,27] reported in the literature suggest a less aggressive surgical option by leaving the endograft in situ, thus performing a semiconversion [31]. It consists of leaving the endograft in situ and opening the aneurysmal sac without the need for aortic clamping, thus minimizing hemodynamic changes and avoiding damage or migration of the endograft. This approach reported mortality ranging from 1 to 4% during total graft removal and 0% in the cases of saccotomy with graft salvage [32,33]. Nevertheless, in our study, no semi-conversions were performed, but 4 total and 2 partial graft explants were performed, with a technical and clinical success rate of 100% in those cases treated in an elective setting.

Furthermore, our data confirms the technical feasibility of a surgical conversion as a definitive treatment for refractory EL2 in an elective setting, but in accordance with previously published experiences, a semiconversion should be considered.

A close follow-up of these patients is therefore recommended.

## 5. Limitations

There are some limitations in our study. First, the retrospective nature of the study was conducted on a non-randomized, single-center reality and a relatively small cohort. Furthermore, although diagnostic images play a crucial role in identifying endoleaks, it is possible that some endoleaks were erroneously categorized. Another limitation of this study was the mean follow-up time, which, although not negligible, could be improved.

Regardless of the limitations, however, identifying patients prone to developing rEL2, which will require an open conversion, is the crucial point.

## 6. Conclusions

Our study supports that EL2, when not resolved at the first attempt, becomes a big concern to resolve. Patients with rEL2 not only with an increased aneurysmal sac but also with stability of the aortic diameter should be considered a high-risk category, with high-risk AAA complications and mortality rate. Our data suggest identifying preoperative variables mainly associated with refractory EL2 to decide the best therapeutic strategy, maybe a more elective aggressive treatment to avoid an urgent intervention.

## Figures and Tables

**Table 1:** Demographics and Comorbidities of the entire study population. CAD: Cardiac Arterial Disease; COPD: chronic obstructive pulmonary disease; EVAR: endovascular aortic repair; IMA: inferior mesenteric artery.

Demographics and Comorbidities	Group A (EL2 Resolution After the First Embolization) Tot. *n*. 19	Group B (Refractory EL2) Tot. *n*. 38	*p*
Mean age (years ± SD)	78.45 ± 7.02	77.43 ± 8.43	0.6540
Male Sex (%, n)	89.4% (17)	92.1% (35)	>0.05
Smoking history	17	29	
Arterial Hypertension (%, n)	73.6% (14)	84.2% (31)	0.49
Dyslipidemia (%, n)	78.9% (15)	84.2% (32)	0.62
CAD (%, n)	26.3% (5)	57.9% (22)	0.002
COPD (%, n)	31.5% (6)	23.7% (9)	0.52
Diabetes Mellitus (%, n)	21.1% (4)	15.8% (6)	0.62
Mean time of EL2 diagnosis (days ± SD)	38 ± 5.2	192 ± 22.7	0.0001
Standard EVAR (%, n)	21.1% (4)	73.7% (28)	0.0001
EVAR + IMA embolization (%, n)	42.10% (8)	5.26% (2)	0.0013
EVAR + Sac embolization (%, n)	36.8% (7)	21% (8)	0.2199
Gore Excluder (%, n)	42% (8)	50% (19)	0.5825
Endurant Medtronic (%, n)	31.6% (6)	26.3% (10)	0.7583
Incraft Cordis (%, n)	15.8% (3)	13.1% (5)	>0.05
Endologix AFX (%, n)	5.3% (1)	5.3% (2)	>0.05
Endologix Ovation (%, n)	5.3% (1)	5.3% (2)	>0.05

**Table 2:** Preoperative aneurysm diameter of more than 65 mm shows seems to be a significant risk factor for the development of refractory Type 2 EL.

Refractory Type 2 EL		
Included Variables	*p*	Error Relative
Preoperative AAA diameter > 65 mm at first EVAR procedure	<0.001	12.95 (34–440.28)
Sac embolization	<0.001	0.182 (6.171–34)
IMA embolization	<0.001	0.055 (1.886–34)

**Table 3:** Aortic transverse diameter revealed at computed tomography angiography images. EVAR: endovascular aortic repair.

	Group A (EL2 Resolution After the First Embolization) Tot. *n* 19	Group B (Refractory EL2) Tot. *n* 38	*p*
Mean aneurysm diameter before EVAR (mm)	54.5 ± 12.06	73.3 ± 10.86	0.0001
Mean aneurysm diameter at first embolization (mm)	63.4 ± 11.23	83.3 ± 12.08	0.0001
Mean aneurysm diameter at second intervention	-	87.2 ± 10.04	

**Table 4:** Anatomical characteristics evaluated at first CTA before EVAR procedure. IMA: inferior mesenteric artery.

Aortic Branches	Group A (EL2 Resolution After the First Embolization) Tot. *n*. 19	Group B (Refractory EL2) Tot. *n*. 38	*p*
IMA patency (n, %)	8 (42.10%)	28 (73.68%)	0.01
Patent lumbar arteries (n, %)			
- 1 couple - 2 couples - 3 couples - >3 couples	2 (10.52%)	2 (5.26%)	0.46
	6 (31.57%)	8 (21.05%)	0.38
	4 (21.05%)	24 (63.1%)	0.002
	1 (5.26%)	12 (31.57%)	0.02

**Table 5:** Type of the first redo embolization procedure. IMA: inferior mesenteric artery.

First Embolization Procedure	Group A (EL2 Resolution After the First Embolization) Tot. *n*. 19	Group B (Refractory EL2) Tot. *n*. 38	*p*
IMA embolization	3 (15.7%)	7 (18.42%)	>0.05
Sac embolization	1 (5.26%)	25 (68.9%)	0.000082
Lumbar embolization	8 (42.1%)	2 (5.26%)	0.00001
Sac + IMA embolization	2 (10.5%)	2 (5.26%)	-
Sac + lumbar embolization	5 (26.3%)	2 (5.26%)	-

## Data Availability

Data are not available due to privacy restrictions.
